# Supplementary material for: Farnesoid X Receptor Agonist GW4064 Protects Lipopolysaccharide-Induced Intestinal Epithelial Barrier Function and Colorectal Tumorigenesis Signaling through the αKlotho/βKlotho/FGFs Pathways in Mice
Source: Int J Mol Sci. 2023 Nov 29;24(23):16932. doi: 10.3390/ijms242316932 (PMC10706872; doi:10.3390/ijms242316932)
Supplement: Supplementary file 1 [file ijms-24-16932-s001.zip › ijms-2713024-supplementary.pdf]

Supplementary Table S1. Scoring parameters of colonic histological changes.

**Score**	**0**	**1**	**2**	**3**	**4**
Inflammation	None	Mild	Moderate	Severe	
Mucosal damage	None	Mucus layer	Submucosa	Muscular and serosa	
Crypt loss	None	1/3	2/3	100 % + intact epithelium	100 % with epithelium lose
Pathological change range	None	1-25 %	26-50 %	51-75 %	76-100 %

Supplementary Table S2. The antibodies used for IF, IHC, and Western blot.

Antibodies	Company/State/ Country	Cat number
ASBT/SLC10A2	Bioss Antibidies, Woburn, MA, USA	Cat# bs-4189R
αKlotho	Abcam, Trumpington, Cambridge, UK	Cat# ab181373
β-actin	Proteintech, Rosemont, IL, USA	Cat# 60008-1-lg
β-catenin	Abcam, Trumpington, Cambridge, UK	Cat# ab6302
βKlotho	GeneTex, Alton Pkwy Irvine, CA, USA	Cat# GTX122197
Caspase 3	Proteintech, Rosemont, IL, USA	Cat# 25546-1-AP
CD133	Abcam, Trumpington, Cambridge, UK	Cat# ab19898
CD34	Abcam, Trumpington, Cambridge, UK	Cat# ab8158
Claudin-1	Abcam, Trumpington, Cambridge, UK	Cat# ab15098
C-Myc	Abcam, Trumpington, Cambridge, UK	Cat# ab32
FGF19	Abcam, Trumpington, Cambridge, UK	Cat# ab225942
FGF21	Abcam, Trumpington, Cambridge, UK	Cat# ab171941
FGF23	Abcam, Trumpington, Cambridge, UK	Cat# ab307421
FXR	Abcam, Trumpington, Cambridge, UK	Cat# ab129089
Histone	Santa Cruz Biotechnology, Santa Cruz, California, USA	Cat# sc-56695
ICAM	Thermo Fisher Scientific, Rockford, IL, USA	Cat# MA5407
LGR5	Abcam, Trumpington, Cambridge, UK	Cat# ab75850
MMP9	Merck Millipore, Burlington, Massachusetts, USA	Cat# NP_004985
MRP2	Thermo Fisher Scientific, Rockford, IL, USA	Cat# MA5-15700
MRP3	Biocompare, South San Francisco, CA, USA	Cat# L1010
MyD88	Abcam, Trumpington, Cambridge, UK	Cat# ab28763
NF-κB	Santa Cruz Biotechnology, Santa Cruz, California, USA	Cat# sc-8008
OATP/SLCO1A2	Merck Millipore, Burlington, Massachusetts, USA	Cat# SAB4502814
OSTβ	Biorbyt, Cambridge, UK	Cat# orb1964
PCNA	Abcam, Trumpington, Cambridge, UK	Cat# ab18197
TGFβRII	Thermo Fisher Scientific, Rockford, IL, USA	Cat# PA5-36115
TLR4	Thermo Fisher Scientific, Rockford, IL, USA	Cat# MA5-16216
VCAM	Abcam, Trumpington, Cambridge, UK	Cat# ab78712
VEGF	Boster Biological Technology, Pleasanton, CA, USA	Cat# PB9071
VEGFR1	Abcam, Trumpington, Cambridge, UK	Cat# ab32152
ZO-1	Abcam, Trumpington, Cambridge, UK,	Cat# ab59720
Goat anti-mouse IgG	MilliporeSigma, Burlington, Massachusetts	Cat# Ap124P
Goat anti-rabbit IgG	Jackson ImmunoResearch Laboratories, West Grove, PA, USA	Cat# 111-065-003
Goat anti-rat IgG	Jackson ImmunoResearch Laboratories, West Grove, PA, USA	Cat# 112-065-003

ASBT/SLC10A2, apical Sodium-dependent Bile acid Transporter; FGF19, fibroblast growth factor 19; FXR, farnesoid X receptor; ICAM, intercellular adhesion molecule; LGR5, leucine-rich repeat-containing G-protein coupled receptor 5; MMP9, matrix metalloproteinase 9; MRP2, multidrug resistance associated protein 2; MyD88, myeloid differentiation primary response protein 88; NF-κB, nuclear factor kappa-light-chain-enhancer of activated B cells; OATP/SLCO1A2, organic-anion-transporting polypeptides; OSTβ, organic solute transporter beta; PCNA, proliferating cell nuclear antigen; TGFβRII, rransforming growth factor beta receptor II; TLR4, toll like receptor 4; VCAM, vascular cell adhesion protein 1;VEGF, vascular endothelial growth factor; VEGFR1, vascular endothelial growth factor receptor 1; ZO-1, zonula occludens 1.

Supplementary Table S3. The sequences of primers used for qRT-PCR.

Gene	Forward Primer sequences (5′ - 3′)	Reverse Primer sequences (5′ - 3′)
Nlrp3	agccttccaggatcctcttc	cttgggcagcagtttctttc
Asc	gaagctgctgacagtgcaac	gccacagctccagactcttc
Panx-1	ggccacggagtatgtgttct	tacagcagcccagcagtatg
Pro-casp 1	agatggcacatttccaggac	gatcctccagcagcaacttc
Tnf-α	ttgacctcagcgctgagttg	cctgtagcccacgtcgtagc
Il-1β	gcaactgttcctgaactca	ctcggagcctgtagtgcag
Ifn-γ	tggcatagatgtggaagaaaagag	tgcaggattttcatgtcaccat
Mrp2	gcttcccatggtgatctctt	atcatcgcttcccaggtact
Mrp3	ctgggtcccctgcatctac	gccgtcttgagcctggataac
Mdr1b	gtgttaaaggggcgatgggcg	aggcttggccagacaacagctt
Mdr2	atcctatgcactggccttctggt	gaaagcatcaatacagggggcag
Asbt	ggaactggctccaatatcctg	gttcccgagtcaacccacat
Ibabp	acaggacttcacctggtc	gcgctcataggtcacatc
Gapdh	tcaccaccatggagaaggc	gctaagcagttggtggtgca
Car	ggaggaccagatctcccttc	atttcattgccactcccaag
Cyp3a11	gtgctcctagcaatcagctt	cagtgcctaaaaatggcagagg
Sult2a1	ggaaggaccacgactcataac	gattcttcacaaggtttgtgttacc
Bcrp	tcgcagaaggagatgtgttgag	ccagaatagcattaaggccagg
Oatp2b1	attgcaggcatcacacaaga	tagaagacctggcttttgcc
Ostα	gccaggcaggactcatatcaaa	ggcaactgagccagtggtaaga
Ostβ	caggaactgctggaagaaatgc	gcaggtcttctggtgtttctttgt
Grp78/Bip	acatggacctgttccgctcta	tggctccttgccattgaaga
Chop	tatctcatccccaggaaacg	gggcactgaccactctgttt
Xbp1s	ctgagtccgaatcaggtgcag	gtccatgggaagatgttctgg
Atf4	atggccggctatggatgat	cgaagtcaaactctttcagatccatt
Lgr5	caagccatgaccttggccctg	tttcccagggagtggattctatt
Atf6	tgggcaggactatgaagtaatg	aggcttggccagacaacagctt
Cyclin D1	ggggacaactcttaagtctcac	ccaataaaagaccaatctctc
Olfm4	cagccactttccaatttcactg	gctggacatactccttcacctta

Nlrp3, NACHT, LRR and PYD domains-containing protein 3; *Asc*, apoptosis-associated speck-like protein containing a caspase recruitment domain; *Tnf-α*, tumor necrosis factor-α; *Il-1β*, interleukin-1β; *Ifn-γ*, Interferon-γ; *Fxr*, farnesoid X receptor; *Atf4*, activating transcription factor; *Grp78*, glucose-regulated protein 78; *Chop*, CCAAT-enhancer-binding protein homologous protein (*Bip*, binding immunoglobulin protein); *Xbp1s*, X-box binding protein 1 Spliced; *Gapdh*, glyceraldehyde 3-phosphate dehydrogenase; *Pxr*, pregnane X receptor; *Car*, constitutive androstane receptor; *Cyp3a11*, Cytochrome P450 3A11; *Sult2a1*, sulfotransferase family 2A member 1; *Mrp2*, multidrug resistance-associated protein 2; *Mdr1b*, multidrug resistance protein; *Bcrp*, breast cancer resistance protein; *Oatp2b1*, organic anion-transporting polypeptide 2B1; *Asbt*, apical sodium dependent bile acid transporter; *Ibabp*, ileal bile acid-binding protein; *Ost*, organic solute transporter; *Lgr5*, Leucine-rich repeat-containing G-protein coupled receptor 5; *Olfm4*, olfactomedin 4.
